# Effects of Air Pollution and Other Environmental Exposures on Estimates of Severe Influenza Illness, Washington, USA

**DOI:** 10.3201/eid2605.190599

**Published:** 2020-05

**Authors:** Ranjani Somayaji, Moni B. Neradilek, Adam A. Szpiro, Kathryn H. Lofy, Michael L. Jackson, Christopher H. Goss, Jeffrey S. Duchin, Kathleen M. Neuzil, Justin R. Ortiz

**Affiliations:** University of Calgary, Calgary, Alberta, Canada (R. Somayaji); University of Washington, Seattle, Washington, USA (R. Somayaji, A.A. Szpiro, C.H. Goss, J.S. Duchin);; The Mountain-Whisper-Light Statistics, Seattle (M.B. Neradilek);; Washington State Department of Health, Shoreline, Washington, USA (K.H. Lofy);; Kaiser Permanente Washington Health Research Institute, Seattle (M.L. Jackson);; Seattle & King County Public Health, Seattle (J.S. Duchin);; University of Maryland School of Medicine, Baltimore, Maryland, USA (K.M. Neuzil, J.R. Ortiz)

**Keywords:** influenza, hospitalization, air pollution, epidemiology, respiratory syncytial virus, RSV, respiratory infections, Washington, United States

## Abstract

Ecologic models of influenza burden may be confounded by other exposures that share winter seasonality. We evaluated the effects of air pollution and other environmental exposures in ecologic models estimating influenza-associated hospitalizations. We linked hospitalization data, viral surveillance, and environmental data, including temperature, relative humidity, dew point, and fine particulate matter for 3 counties in Washington, USA, for 2001–2012. We used negative binomial regression models to estimate the incidence of influenza-associated respiratory and circulatory (RC) hospitalizations and to assess the effect of adjusting for environmental exposures on RC hospitalization estimates. The modeled overall incidence rate of influenza-associated RC hospitalizations was 31/100,000 person-years. The environmental parameters were statistically associated with RC hospitalizations but did not appreciably affect the event rate estimates. Modeled influenza-associated RC hospitalization rates were similar to published estimates, and inclusion of environmental covariates in the model did not have a clinically important effect on severe influenza estimates.

Seasonal influenza is associated with an estimated 3,300–48,000 annual deaths in the United States (*1*) and has a major global impact on economies and health (*2*–*4*). Prospective surveillance with specific laboratory testing for influenza is expensive and may underestimate the true burden of influenza if such tests are underused or insensitive or if influenza results in complications or hospitalizations beyond the period in which virus may be detected in patient samples (*5*). Therefore, the Centers for Disease Control and Prevention (CDC) and other public health organizations use modeling studies to estimate the incidence of severe influenza illness to inform public health actions (*1*,*3*,*6*–*10*). Typically, modeling of the influenza disease burden links aggregate data for outcomes identified in vital statistics or hospitalization administrative databases to influenza virologic surveillance data over time. The difference between estimates with and without influenza covariates is attributed to influenza activity. Such models have been used extensively in the United States (*10*–*14*), in other countries (*15*–*17*), and to produce global estimates of influenza disease burden (*2*,*3*,*18*–*20*). The resulting estimates of excess influenza-associated events inform public health actions, such as vaccine or treatment recommendations and patient and healthcare provider communications.

In the United States, influenza and most other respiratory infections are seasonal and follow an approximately sinusoidal curve with winter peaks. Climatic and air pollutant parameters, such as temperature, humidity, and ambient fine particulate matter, vary during the putative influenza season and are associated with acute respiratory infections (*21*). Because these other factors share a seasonality similar to influenza, neglecting them may overestimate the effects of influenza on health outcomes. Influenza models that include meteorological data have improved predictive accuracy for viral circulation and peak seasonality (*21*–*23*). National and global models of influenza disease burden do not account for environmental and meteorological parameters, which may be important confounding variables (*1*–*3*,*6*–*8*).

Given the importance of influenza disease burden estimates on public health decision making and the reliance on ecologic models for estimation that exclude environmental exposure covariates, we undertook this study to evaluate the effect of including environmental exposures in traditional models on estimates of influenza disease. We hypothesized that environmental exposures would be associated with severe respiratory and circulatory (RC) hospitalizations and that adjustment for these covariates would have a substantial effect on estimates of severe influenza disease incidence.

## Materials and Methods

### Design Overview

We conducted a study using aggregated datasets from 3 counties (King, Pierce, and Snohomish) in western Washington state during 2001–2012. We used administrative hospitalization data, respiratory virus surveillance data, and environmental exposure data collected prospectively from the study area. The primary analysis was to estimate the incidence of influenza-associated RC hospitalizations using standard CDC ecologic models and to assess the effect that inclusion of environmental exposure variables in these models had on the incidence estimates. In a secondary analysis, we added respiratory syncytial virus (RSV) as an additional exposure covariate into the model. This study received exempt review status from the Human Subjects Division at the University of Washington and the Washington State Department of Health Institutional Review Board.

### Hospitalization Database

We obtained the Washington State Department of Health Comprehensive Hospital Abstract Reporting System (CHARS) dataset for the study area and time periods. The CHARS database contains publicly available deidentified discharge information derived from hospital billing systems for patients in all of the public and private hospitals in Washington (*24*). The CHARS data contain information on age, home ZIP code, and other demographics, as well as patient diagnoses, procedures, and billed charges. We defined RC hospitalizations as any listed hospitalizations with codes 390–519 from the International Classification of Diseases, 9th edition(*5*,*6*,*8*). We categorized age as 0–6 months, 7–23 months, 2–4 years, 5–14 years, 15–49 years, 50–64 years, and >65 years. We calculated aggregate RC counts per day, age category, and county and merged them with the other datasets for statistical analysis. The unit of observation was RC hospitalization, which we also call “event” in this report.

### Respiratory Virus Surveillance Data

We accessed influenza virus surveillance data from 3 sources in the study area: University of Washington Clinical Virology Laboratory, Public Health–Seattle & King County, and Seattle Children’s Hospital. Each laboratory was in King County and participated in the United States Influenza Virologic Surveillance System during the study period. Influenza testing data were available for September 30, 2001, through December 29, 2012, except for the third quarter of 2002 (*25*). Clinical specimens collected as part of routine care were tested in laboratories for evidence of influenza virus, and results were reported to local and the state health departments and CDC. The 3 sites used viral culture or reverse transcription PCR (RT-PCR), with an increasing use of RT-PCR over the study period. We did not include influenza data from Tacoma and Snohomish counties. Public health respiratory virus surveillance was not conducted in the counties during the study period. We reviewed limited influenza testing data from the largest hospital systems in each county. Total influenza tests from Tacoma (23,741) and Snohomish counties (<3,000) were very low compared with those from King County (372,022) and were available for only part of our study period (2007–2008 and 2008–2012 for Tacoma and 2010–2013 for Snohomish). Influenza seasonality and peak seasons were similar in all 3 sites. Laboratory reports did not consistently distinguish between influenza A subtypes or influenza B lineages; therefore, we included only influenza A and B as exposure variables. The seasonality and temporal peaks of the proportion positive of influenza A and influenza B data among these sites were similar, so we aggregated each across all 3 counties for analyses.

RSV laboratory data were collected as part of routine care by the University of Washington Clinical Virology Laboratory and reported to the National Respiratory and Enteric Virus Surveillance System; these data were available for the period September 30, 2007–December 29, 2012 (*26*). RSV tests used antigen detection, viral culture, and RT-PCR testing, with RT-PCR use increasing over the period. RSV subtypes were not available.

We used weekly surveillance data for our model. We divided the weekly number of influenza A and influenza B detections by the weekly number of influenza tests performed and multiplied the result by 100 to calculate a weekly percentage of positive tests. We calculated the weekly percentage of positive RSV tests similarly.

### Environmental and Meteorology Exposure Time Series

We accessed daily meteorology data including temperature, relative humidity, and dew point for 6 meteorological stations from each of the 3 counties studied (*27*,*28*) (Appendix Figures 1–5). The values for the 6 stations were highly correlated (Pearson correlation coefficient range 0.93–0.99). Because the data from Boeing Field station in King County were the most complete and the station was the closest to the urban centers, we used data from this station to represent the meteorological exposures for the entire study area. For the meteorological data, including temperature, relative humidity, and dew point, 680 weeks (4,736 days) of data were available, leaving <0.005% of days with missing data during the study period. 

We also used daily ambient outdoor air pollution data in the form of concentration of particulate matter with a diameter <2.5 μm (PM_2.5_) (*29*,*30*) (Appendix Figures 6, 7). The PM_2.5_ concentration data were available for 21 stations from each of the 3 counties, giving a total of 4,581 days of data. Some of the stations were distant from urban centers (e.g., the Mount Rainier National Park station in Pierce County); others had substantial periods with missing data during the study period. Three stations, Seattle–Beacon Hill (King County), Tacoma (Pierce County), and Marysville (Snohomish County), were close to urban centers and had less missing data; we used these sites to define the daily PM_2.5_ exposures. Because the daily PM_2.5_ exposures for the 3 stations were highly correlated with only small systematic differences (Pearson correlation coefficients among pairs of stations 0.74–0.91), we averaged daily PM_2.5_ exposures across the available values for the stations. The resulting daily average was available in 96% of the study period.

### Population Estimates

We obtained annual age-specific population estimates for each of the 3 counties for 2001–2012 from Washington State Office of Financial Management (OFM) (*30*). OFM population estimates for 2000–2010 are based on the 2000 and 2010 US Census and an interpolation in the intermediate years (*31*). OFM population estimates for 2011 and 2012 were developed using the component method, which derives the estimated population by adding natural population change (births minus deaths) and net migration to the base-year population (*32*). Population estimates for the 0–6 month, 7–23 month, and 2–4 year age groups were not available from OFM data and were estimated from the annual birth data from the Washington State Department of Health (*32*). W carried the annual birth numbers for each county forward in time to estimate the population sizes for the 0–6 month, 7–23 month, and 2–4 year categories at a specific time point. Because births were reported annually and our age categories included half-year fractions (0–6 month and 7–23 month), we used halves of the appropriate annual birth numbers to estimate population sizes in these age categories.

### Statistical Analysis

To describe all-cause RC hospitalizations, we calculated rates of any RC hospitalization divided by person-time under observation for each age category. To estimate influenza-associated events, we adapted negative binomial regression models used previously by CDC to estimate the incidence of influenza-associated hospitalizations from surveillance data and administrative hospitalization datasets (*5*,*6*,*8*,*33*,*34*). We fitted age-specific negative binomial regression models to daily events in the 3 counties of interest (Appendix). Covariates were time (day expressed as a fraction of the year), daily RC hospitalizations in a particular county on a particular day, the county’s population size in that calendar year, the percentage of specimens testing positive in the corresponding week for influenza A and influenza B, daily environmental effects, and terms accounting for secular and seasonal trends. The offsets for county and population in the model account for different population sizes across counties and years. The environmental effects include the effect of the temperature, humidity, dew point, and PM_2.5_ concentration. We modeled the effect of each of these 4 variables by exposure on the same day and by exposure on the previous day (one-day lag term). We used a cubic b-spline with 3 degrees of freedom for both the same day and 1-day lag terms for a total of 24 adjustment coefficients for the 4 environmental variables.

For each age category, we fit a base model, which was similar to CDC ecologic models and excludes environmental exposures, and an expanded model, which included the environmental exposures. Using each fitted model, we calculated the number of age-specific influenza-associated RC hospitalizations as the difference between model-predicted RC hospitalizations estimated from the original data and model-predicted RC hospitalizations with all influenza terms set to 0. We calculated the number of type-specific influenza-associated RC hospitalizations (influenza A or B) in a similar fashion but by setting only one of the influenza terms to 0. To express the influenza-associated RC hospitalizations as rates, we divided them by the age-specific population estimates (presented as the number of events per 10,000 person-months or 100,000 person-years). We calculated population-attributable risks (PARs) for influenza-associated RC hospitalizations for each age category as the number of influenza-associated RC hospitalizations divided by the number of all-cause RC hospitalizations. We calculated 95% CIs for the number of influenza-associated RC hospitalizations, rates, and PARs using the nonparametric bootstrap (*35*).

To assess the effect of inclusion of RSV in our models, our secondary analysis expanded the model by adding an additional term, β_35_RSV, for the effect of the percentage of specimens testing positive in the corresponding week for RSV. We calculated the numbers of virus-specific (disaggregated) and the total (influenza + RSV) attributable RC hospitalizations as the corresponding rates and PARs. We calculated incidence rates for RSV-associated outcomes similarly to the influenza outcomes. We limited the fit of the RSV model to the period of RSV data availability.

We conducted 3 sensitivity analyses based on the primary analysis model to assess the effect of alternative modeling choices: 1) analysis with the environmental exposure modeled as linear instead of as the cubic b-spline (data not shown); 2) analysis without the 1-day-lag environmental exposure variables; and 3) analysis with weekly events instead of daily events. We compared the results of the primary analysis to these alternative modeling choices and found no major differences. Statistical diagnostics of the models included added variable plots and likelihood ratio tests for distributed lags (day 2 through day 6 lags) and illustrated adequate model fit. We performed analyses with R version 3.1.0 statistical software (https://r-project.org).

## Results

The study populations ranged from 1,758,779 (King), 708,230 (Pierce), and 615,435 (Snohomish) in 2001 to 1,960,782 (King), 808,316 (Pierce), and 723,301 (Snohomish) in 2012. A total of 1,503,081 all-cause RC hospitalizations occurred in these 3 counties during September 30, 2001–December 29, 2012, for an overall incidence rate of 4,600/100,000 person-years. Incidence rates were highest at the extremes of age (0–6 months, 5,949/100,000 person-years; 50–64 years, 6,503/100,000 person-years; and >65 years, 23,077/100,000 person-years).

**Figure 1:**
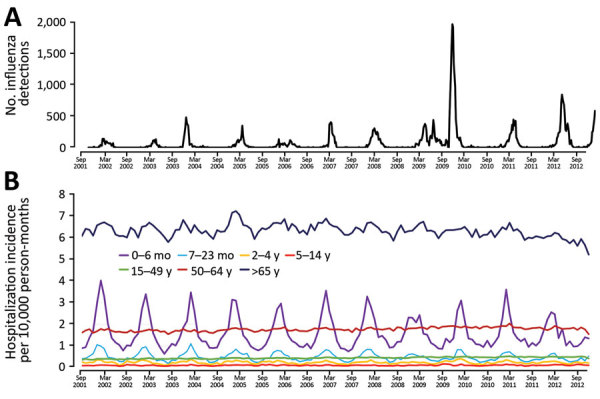
Influenza detections and respiratory and circulatory hospitalizations in western Washington, USA, 2001–2012. A) Total detections of influenza by clinical laboratories and public health surveillance. B) Incidence of all-cause respiratory and circulatory hospitalizations by age group..

Using the base model, incorporating time and seasonality, and excluding environmental exposures, the overall incidence rate of influenza-associated RC hospitalizations was 31/100,000 person-years with 0.7% PAR. Event rates varied across age groups and had a marked winter seasonality over the study period (Figure 1). In the base model, influenza-attributable events rate was highest in the 0–6 months age group (118.7/100,000 person-years) and the >65 years age group (157.0/100,000 person-years). Of these, the influenza A attributable event rate was highest in the same 2 age groups (0–6 months, 159.9/100,000 person-years; >65 years, 81.3/100,000 person-years), and influenza B rate was highest in the >65 years age group (76.2/100,000 person-years) (Table 1). Overall, influenza A was associated with higher hospitalization rates than influenza B (21.3 vs. 10.3/100,000 person-years).

**Table 1:** Influenza-associated respiratory and circulatory hospitalizations by age group modeled with and without environmental covariates, October 2001–December 2012*

Model type and age group	All influenza-attributable events	All influenza-attributable events/100,000 person-years	Influenza A–attributable events†	Influenza A–attributable events/100,000 person-years†	Influenza B–attributable events	Influenza B– attributable events/100,000 person-years
Without environmental covariates‡						
0–6 mo	254	118.7	342	159.9	−92	−42.9
7–23 mo	88	13.8	176	27.6	−90	−14.1
2–4 y	218	17.5	242	19.4	−25	−2.0
5–14 y	735	17.5	547	13.0	199	4.7
15–49 y	2,108	12.4	1,835	10.8	276	1.6
50–64 y	2,204	37.3	1,849	31.3	358	6.1
>65 y	5,376	157.0	2,782	81.3	2,609	76.2
With environmental covariates‡						
0–6 mo	239	111.9	308	143.9	−71	−33.2
7–23 mo	87	13.6	174	27.2	−89	−13.9
2–4 y	215	17.2	240	19.3	−26	−2.1
5–14 y	741	17.6	571	13.6	181	4.3
15–49 y	1,885	11.1	1,618	9.5	270	1.6
50–64 y	2,064	34.9	1,667	28.2	401	6.8
>65 y	5,043	147.3	2,371	69.3	2,685	78.4

*The number of all influenza-attributable events is not equal to the sum of influenza A and influenza B events because the 2 types of influenza exposure are not independent and their attribution can overlap.  †We could not discern between influenza A(H3N2) and influenza A(H1N1) because of testing limitations over the study period.  ‡Environmental covariates included daily averages of temperature, relative humidity, dew point, and particulate matter with a diameter <2.5 μm.

**Figure 2:**
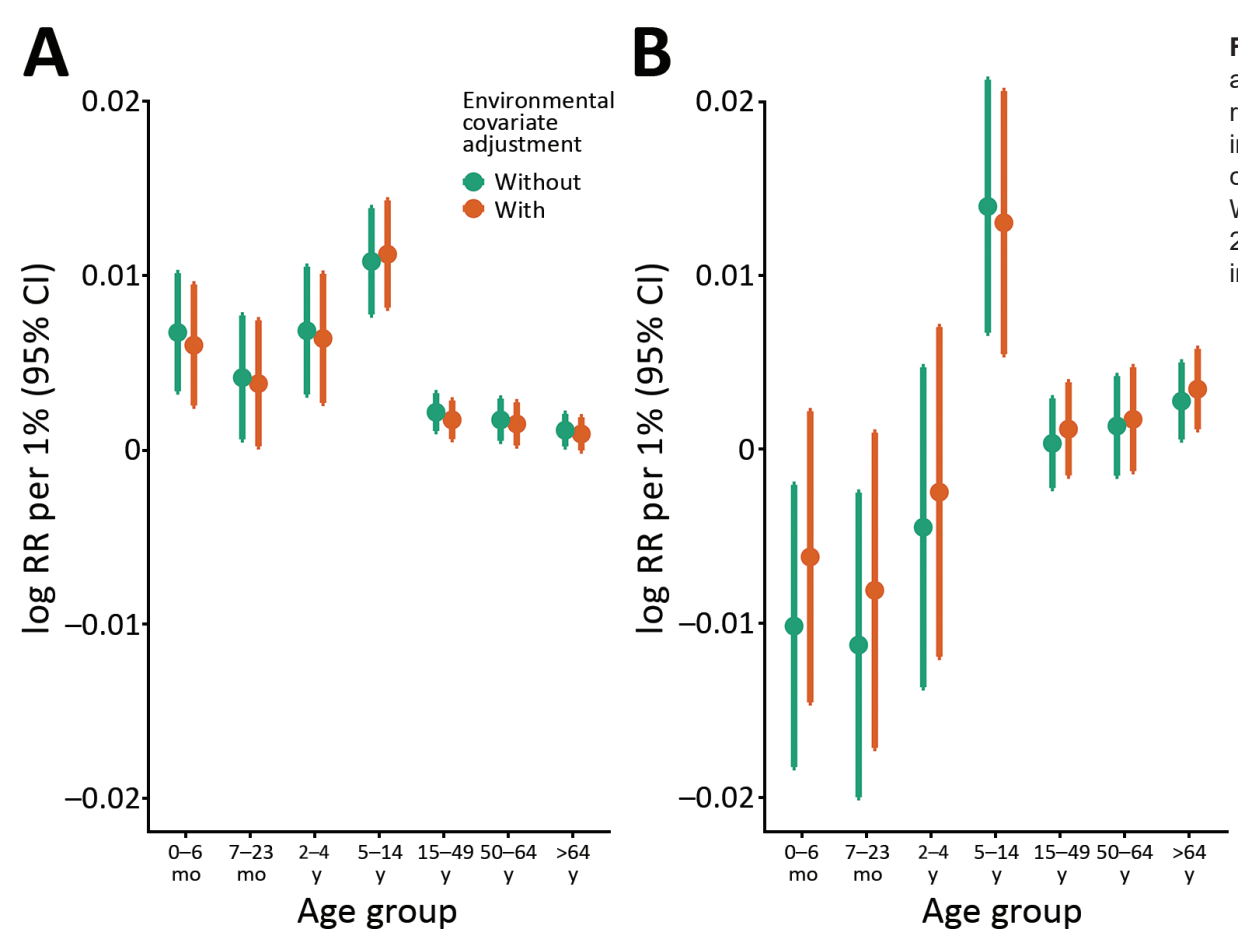
Influenza-associated hospitalization risk by age, with and without inclusion of environmental covariates, western Washington, USA, 2001–2012. A) Influenza A; B) influenza B.

In the expanded model incorporating environmental covariates, the air pollution and meteorological covariates were strongly associated with RC hospitalizations (Appendix Table 2); however, the influenza-associated event rates did not change appreciably in any age group (Table 1; Figure 2). The overall influenza-attributable rate was similar at 31.4/100,000 person-years (influenza A, 21.3/100,000 person-years; influenza B, 10.3/100,000 person-years). The influenza-associated event rates were highest in the 0–6 month (111.9/100,000 person-years) and the >65 years (147.3/100,000 person-years) age groups. PAR was highest in the 5–14 years age group (4.8%; 95% CI 3.7%–6.0%) (Table 2). When assessed by influenza type, influenza A had a greater number of attributable events in all age groups with the exception of the >65 years age group (influenza A, 69.3/100,000 person-years; influenza B, 78.4/100,000 person-years). Similarly, PAR was greater for influenza A across age groups with the exception of the >65 years group (Table 2). PAR for influenza was similar in both the base (without environmental covariates) and expanded (with environmental covariates) models.

**Table 2:** Population attributable risk of influenza-associated respiratory and circulatory hospitalizations by age group modeled with and without environmental covariates, October 2001–December 2012

Model type	Age group						
	0–6 mo	7–23 mo	2–4 y	5–14 y	15–49 y	50–64 y	≥65 y
Without environmental covariates*							
All influenza, %	2.0 (0.6–3.4)	0.8 (−0.8 to 2.3)	2.0 (0.6–3.4)	4.8 (3.7–6.0)	0.8 (0.3–1.1)	0.6 (0.2–1.0)	0.7 (0.4–1.0)
Influenza A, %†	2.7 (1.4–4.0)	1.5 (0.2–2.9)	2.3 (1.0–3.5)	3.6 (2.5–4.7)	0.7 (0.3–1.0)	0.5 (0.1–0.8)	0.4 (0.1–0.6)
Influenza B, %	−0.7 (−1.6 to 0.2)	−0.8 (−1.6 to 0.1)	−0.2 (−1.1 to 0.6)	1.3 (0.7–1.9)	0.1 (−0.1 to 0.3)	0.1 (−0.1 to 0.3)	0.3 (0.1–0.5)
With environmental covariates*							
All influenza, %	1.9 (0.5–3.3)	0.8 (−0.8 to 2.2)	2.0 (0.6–3.4)	4.8 (3.7–6.0)	0.7 (0.3–1.0)	0.5 (0.1–0.9)	0.6 (0.3–1.0)
Influenza A, %†	2.4 (1.2–3.7)	1.5 (0.1–2.9)	2.3 (1.0–3.5)	3.7 (2.6–4.8)	0.6 (0.2–0.9)	0.4 (0.0–0.8)	0.3 (−0.0 to 0.6)
Influenza B, %	−0.6 (−1.4 to 0.4)	−0.8 (−1.6 to 0.1)	−0.2 (−1.1 to 0.6)	1.2 (0.5–1.8)	0.1 (−0.1 to 0.3)	0.1 (−0.1 to 0.3)	0.3 (0.1–0.5)
Difference‡							
All influenza	−0.1	0	0	0	−0.1	−0.1	−0.1
Influenza A†	−0.3	0	0	0.1	−0.1	−0.1	−0.1
Influenza B	0.1	0	0	−0.1	0	0	0

*Environmental covariates included daily averages of temperature, relative humidity, dew point, and particulate matter with a diameter <2.5 μm. †We could not discern between influenza A(H3N2) and influenza A(H1N1) because of testing limitations over the study period.  ‡With covariates minus without covariates.

### Secondary Analysis—Influenza and RSV Models

Among data with other covariates available, RSV data were available for 1,811 days and were analyzed with influenza in models with and without environmental covariates (Appendix Table 1). In the base model incorporating time and seasonality, similar to influenza, RSV-attributable event rates were highest in the youngest and oldest age groups. In the expanded model incorporating environmental covariates, the attributable event rates for influenza or RSV did not appreciably change. PAR for influenza- or RSV-associated RC hospitalizations in the expanded model was 1.0%–12.9% and did not differ from the base model results (Table 3).

**Table 3:** Secondary analysis of population attributable risk (PAR) of influenza or respiratory syncytial virus (RSV)-associated respiratory and circulatory hospitalizations by age group modeled with and without environmental covariates, September 2007–December 2012

Model type	Age group						
	0–6 mo	7–23 mo	2–4 y	5–14 y	15–49 y	50–64 y	≥65 y
Without environmental covariates*							
All influenza and RSV, %	14.0 (10.0–17.6)	6.5 (2.0–10.6)	6.9 (2.6–10.9)	8.3 (5.1–11.3)	1.2 (0.2–2.3)	1.4 (0.1–2.6)	0.6 (−0.4 to 1.5)
Influenza A,† %	1.9 (−0.1 to 3.9)	2.7 (0.4–5.0)	2.7 (0.5–4.6)	5.8 (4.0–7.5)	0.7 (0.2–1.2)	0.6 (−0.0 to 1.1)	0.0 (−0.5 to 0.5)
Influenza B, %	−0.4 (−1.9 to 1.0)	−0.9 (−2.5 to 0.6)	0.2 (−1.1 to 1.4)	1.7 (0.7–2.7)	−0.0 (−0.3 to 0.3)	0.2 (−0.1 to 0.6)	0.4 (0.1–0.7)
RSV, %	12.8 (9.4–15.8)	4.8 (1.0–8.0)	4.2 (0.9–7.6)	1.1 (−1.8 to 3.8)	0.5 (−0.3 to 1.3)	0.6 (−0.4 to 1.6)	0.2 (−0.6 to 1.0)
With environmental covariates*							
All influenza and RSV, %	12.9 (8.6–16.7)	6.9 (2.3–10.9)	7.4 (3.0–11.6)	10.2 (6.9–13.4)	1.7 (0.6–2.7)	1.9 (0.6–3.1)	1.0 (0.0–2.0)
Influenza A,† %	2.0 (0.0–4.0)	2.8 (0.4–5.0)	3.1 (1.0–5.0)	6.1 (4.3–7.8)	0.6 (0.1–1.1)	0.5 (−0.1 to 1.0)	−0.1 (−0.6 to 0.4)
Influenza B, %	−0.4 (−1.8 to 1.0)	−1.0 (−2.6 to 0.6)	−0.0 (−1.3 to 1.2)	1.6 (0.6–2.6)	−0.0 (−0.3 to 0.3)	0.2 (−0.1 to 0.5)	0.4 (0.1–0.7)
RSV, %	11.5 (8.1–14.6)	5.1 (1.3–8.6)	4.4 (0.8–8.2)	2.8 (−0.1 to 5.6)	1.1 (0.2–2.0)	1.2 (0.1–2.2)	0.7 (−0.1–1.5)
Risk difference‡							
All influenza	−1.1	0.4	0.5	1.9	0.5	0.5	0.4
Influenza A†	0.1	0.1	0.4	0.3	−0.1	−0.1	−0.1
Influenza B	0	−0.1	−0.2	−0.1	0	0	0
RSV	−1.3	0.3	0.2	1.7	0.6	0.6	0.5

*Environmental covariates included daily averages of temperature, relative humidity, dew point, and particulate matter with a diameter <2.5 μm. †We could not discern between influenza A(H3N2) and influenza A(H1N1) because of testing limitations over the study period.  ‡With covariates minus without covariates.

### Sensitivity Analysis—Examining Alternative Modeling Choices

We performed sensitivity analysis using alternative modeling choices: environmental covariates modeled as linear; environmental covariates modeled without lag terms; and models run on weekly aggregates. We ran these alternative models for influenza alone (primary analysis) and for influenza with RSV (secondary analysis). We found, as in our initial models, that environmental parameters were strongly associated with RC hospitalizations. However, we saw no meaningful changes in the attributable event rates (Appendix Figures 8, 9). 

## Discussion

We conducted a population-based study incorporating hospitalization, laboratory, and meteorological data from 3 Washington counties over 12 years to estimate the burden of influenza- and RSV-associated RC hospitalizations. Hospitalization rates peaked during the winter, corresponding to periods of influenza circulation, and the highest rates were at the extremes of age for both influenza and RSV events. Our overall influenza-associated hospitalization rate estimate was 31/100,000 years. This is similar to CDC estimates of 55.0/100,000 person-years (95% CI 22.5–125.4) over a period including the 1990s, which was notable for high rates of severe influenza (*6*,*7*). Our age-specific models demonstrated that factors of temperature, relative humidity, dew point, and particulate matter were strongly associated with RC hospitalizations. However, the inclusion of environmental covariates did not result in clinically meaningful changes in respiratory virus–associated event estimates. In addition, we conducted several alternative models, including linear adjustments for environmental parameters (instead of cubic-splines), models without lags for environmental parameters (versus models with lags), and models on weekly-aggregated data (versus daily-aggregated), which led to the same conclusions and added confidence to our results.

Several environmental parameters have been found to improve forecasts of influenza activity, and they may contribute to influenza illness in several ways. Climatic variables, such as temperature and humidity, increase the survival and spread of influenza in the environment (*23*,*36*). These same factors change human behaviors and enhance virus transmission by driving people indoors and increasing crowding. Air pollution increases every winter and is strongly associated with respiratory infections (*37*). Certain climate conditions, including temperature, humidity, and particulate matter can affect susceptibility to upper respiratory infections (*38*–*41*). Despite these well-known associations between environmental exposures and respiratory events, our study found that their inclusion in a model designed to estimate influenza illness in western Washington had a negligible effect. Whether their influence on disease burden estimates remains small in regions with more extreme weather or air pollution is unclear.

Our study should be interpreted in light of its strengths and limitations. Because our data were from western Washington state, the study may not be generalizable to other regions in which environmental factors may differ. Our focus on a limited geographic area can increase confidence that the population studied was truly exposed to the environmental covariates used in our models, but this design choice limits our ability to evaluate rarer outcomes, such as critical illness or death. We used clinical and virologic surveillance data to model the incidence rates for severe influenza but did not have specific data relating to influenza vaccine, and we were not able to incorporate other respiratory viruses because we lacked robust surveillance data for the study period. For influenza and RSV, the use of percent positive rather than absolute numbers in the model corrects for changing surveillance intensity over time but may decrease estimates of disease incidence during intense seasons when testing volume also increases. We did not have subtype information for influenza or RSV available, which limited our ability to assess whether certain circulating strains were more affected by environmental covariates. Of the meteorological and pollution factors, we did not assess absolute humidity, wind velocity, sunshine duration, ozone, or other measures of pollution, and it is possible that one or more of these factors either independently or in addition may have modified the effect on influenza- or RSV-associated RC hospitalizations. Finally, this is an ecologic study, and the results may not necessarily be representative of patient-level associations. Regardless, this comprehensive study spans over a decade of data using expanded standard ecologic models to assess the relationships of respiratory virus–associated hospitalizations and meteorological and pollution variables in a large population comprising children and adults.

In conclusion, our population-based study in western Washington state over 12 years assessed how incorporation of environmental and air pollution covariates can influence influenza- and RSV-associated disease burden estimates. Our modeled estimates for influenza and RSV hospitalization rates were similar to national rates and changed little with incorporation of seasonal environmental covariates. Our study should strengthen confidence in the traditional ecologic models used to estimate influenza illness. In addition to continued efforts to reduce the extent of vaccine-preventable respiratory viral disease, future work should assess the role environmental parameters have on severe influenza and RSV outcomes in regions with more extreme pollution and meteorological exposures. Finally, there is ample evidence that environmental pollution is deleterious to health, irrespective of its impact on influenza, and more needs to be done to improve the quality of air we breathe.

Appendix Additional information on the impact of air pollution and other environmental exposures on estimates of severe influenza illness.
