# Supplementary material for: Effects of Air Pollution and Other Environmental Exposures on Estimates of Severe Influenza Illness, Washington, USA
Source: Emerg Infect Dis. 2020 May;26(5):920–9. doi: 10.3201/eid2605.190599 (PMC7181929; doi:10.3201/eid2605.190599)
Supplement: Appendix — Additional information on the impact of air pollution and other environmental exposures on estimates of severe influenza illness. [file 19-0599-Techapp-s1.pdf]

Article DOI: https://doi.org/10.3201/eid2605.190599

# Effects of Air Pollution and Other Environmental Exposures on Estimates of Severe Influenza Illness, Washington, USA

## Appendix

### Extended Description of Statistical Analysis

We adapted negative binomial regression models used previously by the US Centers for Disease Control and Prevention to estimate the incidence of influenza-associated hospitalizations from surveillance data and administrative hospitalization datasets (*1–5*). We fit age-specific negative binomial regression models to daily events in the 3 counties of interest. The model is as follows:

$$\log(E(Y_t)) = \log(\alpha_t) + \beta_0 + \beta_1 t + \beta_2 t^2 + \beta_3 t^3 + \beta_4 t^4 + \beta_5 \sin(2\pi t) + \beta_6 \cos(2\pi t) + \beta_7 A + \beta_8 B + \beta_9 PI + \beta_{10} SN + \beta_{11} Z_{11} + \ldots + \beta_{34} Z_{34}$$

where t is the day expressed as a fraction of the year; $Y_t$ represents the number of daily RC hospitalizations in a particular county on a particular day; $\alpha_t$ is equal to the county's population size in that calendar year, $\beta_1$ through $\beta_4$ account for secular trend; $\beta_5$ through $\beta_6$ account for seasonal trend, $\beta_7$ and $\beta_8$ represent the effect of the percentage of specimens testing positive in the corresponding week for influenza A ($\beta_7$) and influenza B ($\beta_8$); $\beta_9$ and $\beta_{10}$ account for the county (King County is the reference); and $\beta_{11}$ through $\beta_{34}$ account for the daily environmental effects. The offsets ($\alpha_t$) for county and population in the model account for different population sizes across counties and years. The environmental effects include the effect of temperature, humidity, dew point, and $PM_{2.5}$ concentration. The effect of each of these 4 variables is modeled by the exposure on the same day and by the exposure on the previous day (1-day lag term). A cubic b-spline with 3 degrees of freedom is used for both the same-day and 1-day lag terms, leading to a total of 24 adjustment coefficients ($\beta_{11}$ through $\beta_{34}$) for the 4 environmental variables. Separate models were fit for each age category.

For each age category, we fit a base model, which is similar to CDC models and excludes environmental exposures (the equation above without environmental terms), and an expanded model, which included the environmental exposures (represented by the full equation above). Using each fitted model, we calculated the number of the age-specific influenza-associated RC hospitalizations as the difference between the model-predicted RC hospitalizations estimated from the original data and the model-predicted RC hospitalizations with all influenza terms set to zero. We calculated the number of type-specific influenza-associated RC hospitalizations (influenza A or B) in a similar fashion but by setting only one of the influenza terms to zero. To express the influenza-associated RC hospitalizations as rates, we divided them by the age-specific population estimates (presented as the number of events per 10,000 person-months or 100,000 person-years). We calculated influenza-associated population-attributable risks (PARs) for influenza-associated RC hospitalizations for each age category as the number of influenza-associated RC hospitalizations divided by the number of all-cause RC hospitalizations. We calculated 95% CI for the number of influenza-associated RC hospitalizations, rates, and PARs using the nonparametric bootstrap (*6*).

To assess the effect of inclusion of RSV in our models our secondary analysis expanded the model by adding an additional term $\beta_{35}$RSV for the effect of the percentage of specimens testing positive in the corresponding week for RSV. We calculated the numbers of virus-specific (disaggregated) and the total (influenza+RSV) attributable R&C hospitalizations as the corresponding rates and PARs. The fit of the RSV model was limited to the period of RSV data availability.

We conducted 3 sensitivity analyses based on the primary analysis model to assess the effect of alternative modeling choices: 1) analysis with the environmental exposure modeled as linear instead of the cubic b-spline; 2) analysis without the 1-day lag environmental exposure variables; and 3) analysis with weekly events instead of daily events. We compared the results of the primary analysis to these alternative modeling choices and found no major differences. Statistical diagnostics of the models included added variable plots and likelihood ratio tests for distributed lags (day 2–day 6 lags) and illustrated adequate model fit.

We performed analyses with R statistical software version 3.1.0 (https://r-project.org).

**References**

<jrn>1. Ortiz JR, Neuzil KM, Cooke CR, Neradilek MB, Goss CH, Shay DK. Influenza pneumonia surveillance among hospitalized adults may underestimate the burden of severe influenza disease. PLoS One. 2014;9:e113903. PubMed https://doi.org/10.1371/journal.pone.0113903</jrn>

<jrn>2. Thompson WW, Shay DK, Weintraub E, Brammer L, Bridges CB, Cox NJ, et al. Influenza-associated hospitalizations in the United States. JAMA. 2004;292:1333–40. PubMed https://doi.org/10.1001/jama.292.11.1333</jrn>

<jrn>3. Zhou H, Thompson WW, Viboud CG, Ringholz CM, Cheng PY, Steiner C, et al. Hospitalizations associated with influenza and respiratory syncytial virus in the United States, 1993–2008. Clin Infect Dis. 2012;54:1427–36. PubMed https://doi.org/10.1093/cid/cis211</jrn>

<jrn>4. Ortiz JR, Neuzil KM, Rue TC, Zhou H, Shay DK, Cheng PY, et al. Population-based incidence estimates of influenza-associated respiratory failure hospitalizations, 2003 to 2009. Am J Respir Crit Care Med. 2013;188:710–5. PubMed https://doi.org/10.1164/rccm.201212-2341OC</jrn>

<jrn>34. Ortiz JR, Neuzil KM, Shay DK, Rue TC, Neradilek MB, Zhou H, et al. The burden of influenza-associated critical illness hospitalizations. Crit Care Med. 2014;42:2325–32. PubMed https://doi.org/10.1097/CCM.0000000000000545</jrn>

<bok>6. Davison AC, Hinkley DV. Bootstrap methods and their application. Cambridge: Cambridge University Press; 1997.</bok>

**Appendix Table 1.** Secondary analysis of influenza- and RSV-associated respiratory and circulatory hospitalizations by age group modeled with and without environmental covariates, October 2001–December 2012*

| Model type | Age category | All influenza and RSV attributable events | All influenza and RSV attributable events/100,000 person-years | Influenza A attributable events | Influenza A attributable events/100,000 person-years | Influenza B attributable events | Influenza B attributable events/100,000 person-years | RSV attributable events | RSV attributable events/100,000 person-years |
|---|---|---|---|---|---|---|---|---|---|
| Model without environmental covariates | 0–6 mo | 915 | 813.1 | 124 | 109.9 | –27 | –24.3 | 833 | 740.1 |
| | 7–23 mo | 366 | 108.8 | 154 | 45.7 | –52 | –15.6 | 269 | 79.8 |
| | 2–4 y | 384 | 58.9 | 149 | 22.8 | 10 | 1.5 | 234 | 35.9 |
| | 5–14 y | 663 | 31.2 | 462 | 21.7 | 136 | 6.4 | 84 | 4.0 |
| | 15–49 y | 1,811 | 20.9 | 1,046 | 12.0 | –3 | 0.0 | 775 | 8.9 |
| | 50–64 y | 2,962 | 90.8 | 1,218 | 37.3 | 486 | 14.9 | 1274 | 39.1 |
| | >64 y | 2,523 | 137.0 | 46 | 2.5 | 1,676 | 91.0 | 804 | 43.7 |
| Model with environmental covariates | 0–6 mo | 839 | 744.9 | 128 | 113.4 | –28 | –24.5 | 752 | 667.7 |
| | 7–23 mo | 387 | 115.0 | 160 | 47.6 | –57 | –16.9 | 288 | 85.5 |
| | 2–4 y | 408 | 62.6 | 171 | 26.2 | 0 | 0.0 | 246 | 37.7 |
| | 5–14 y | 812 | 38.2 | 489 | 23.0 | 129 | 6.1 | 226 | 10.6 |
| | 15–49 y | 2,542 | 29.3 | 946 | 10.9 | –37 | –0.4 | 1,645 | 18.9 |
| | 50–64 y | 4,119 | 126.2 | 1,055 | 32.3 | 453 | 13.9 | 2,636 | 80.8 |
| | >64 y | 4,339 | 235.6 | –340 | –18.5 | 1,584 | 86.0 | 3,104 | 168.6 |

*We could not discern between influenza A H3N2 and influenza A H1N1 due to testing limitations over the study period. All events totals do not equal the sum of RSV–influenza A and influenza B events–as the 3 exposures are not independent (and their attribution can overlap). Environmental covariates included daily averages of temperature, relative humidity, dew point, and particulate matter with a diameter of less than 2.5 μm ($PM_{2.5}$).

**Appendix Table 2.** Effects of environmental and air pollution parameters, expressed as p values, on respiratory and circulatory hospitalizations in the expanded multivariate models*

| Age category | Temperature (6 d.f.) | Relative humidity (6 d.f.) | Dew point (6 d.f.) | Temperature + relative humidity + dew point (18 d.f.) | $PM_{2.5}$ (6 d.f.) | All environmental and air pollution parameters (24 d.f.) |
|---|---|---|---|---|---|---|
| 0-6 mo | 0.3 | 0.5 | 0.08 | <0.001 | 0.02 | <0.001 |
| 7-23 mo | 0.7 | 0.3 | 0.7 | 0.2 | 0.08 | 0.01 |
| 2-4 y | 0.1 | 0.4 | 0.2 | 0.002 | 0.3 | <0.001 |
| 5-14 y | 0.12 | 0.5 | 0.4 | <0.001 | 0.3 | <0.001 |
| 15-49 y | 0.3 | 0.09 | 0.2 | 0.047 | <0.001 | <0.001 |
| 50-64 y | 0.2 | 0.2 | 0.003 | 0.003 | 0.2 | 0.001 |
| >64 y | 0.08 | 0.08 | 0.01 | <0.001 | 0.09 | <0.001 |

*Likelihood ratio test. In the models each parameter (e.g., temperature) was represented by 6 terms (3 terms for the no-lag spline and 3 terms for the lag spline). The p values represent the joint test of coefficients for all relevant terms.

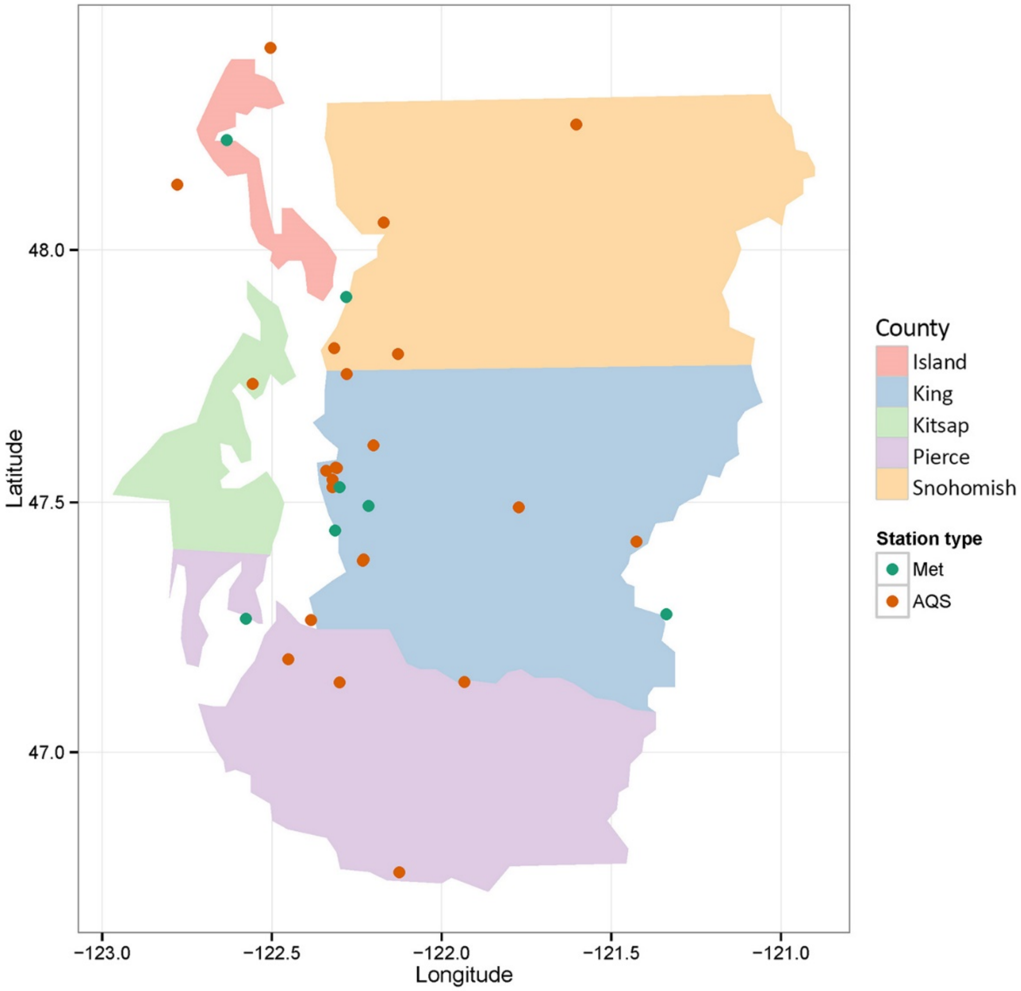


**Appendix Figure 1.** Meteorological (Met) and air quality system (AWS) stations in western Washington State, 2001–2012.

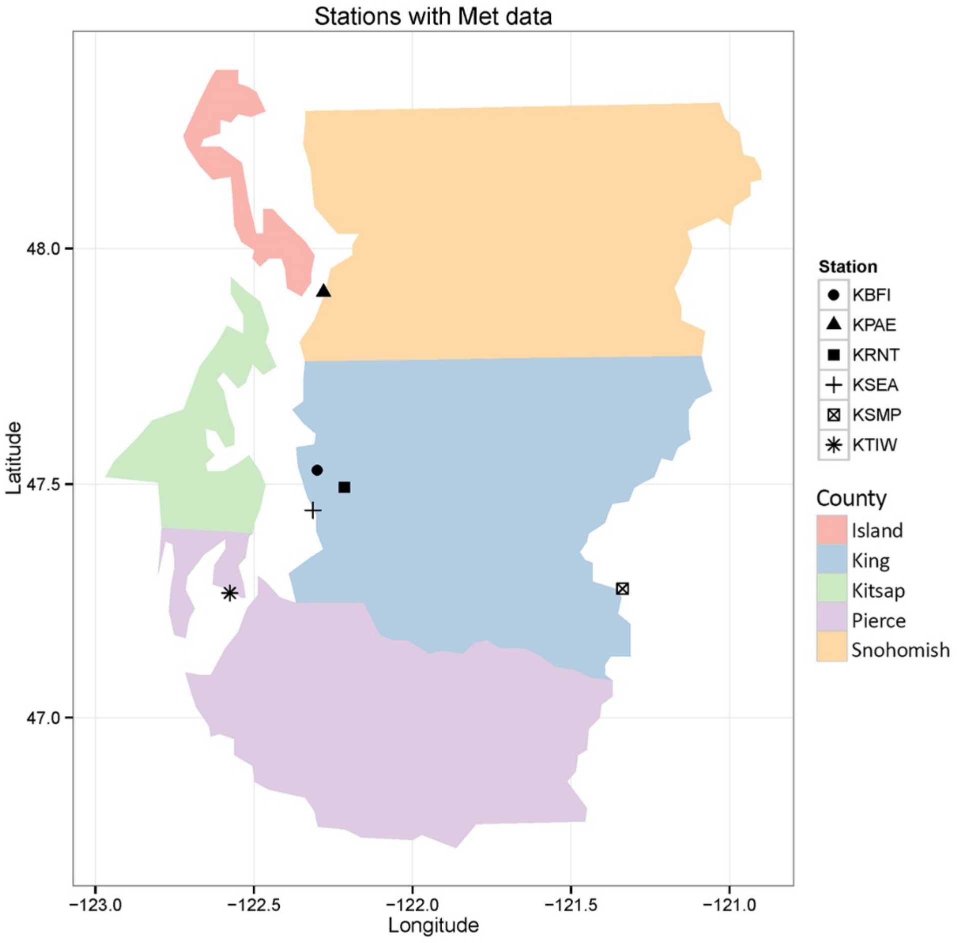


**Appendix Figure 2.** Meteorological (Met) stations in western Washington State, 2001–2012.

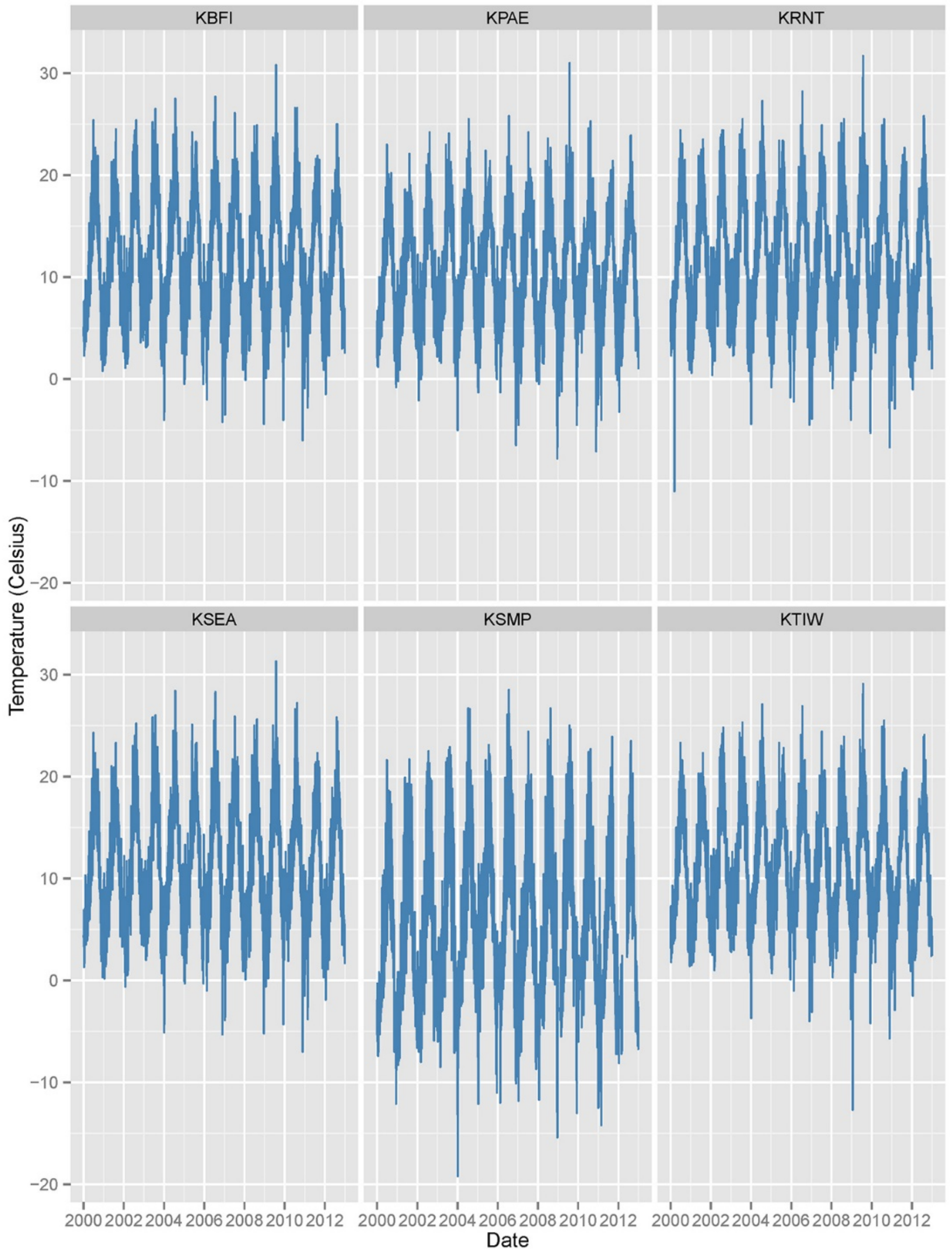


**Appendix Figure 3.** Measured temperature by meteorological stations in western Washington State, 2001–2012.

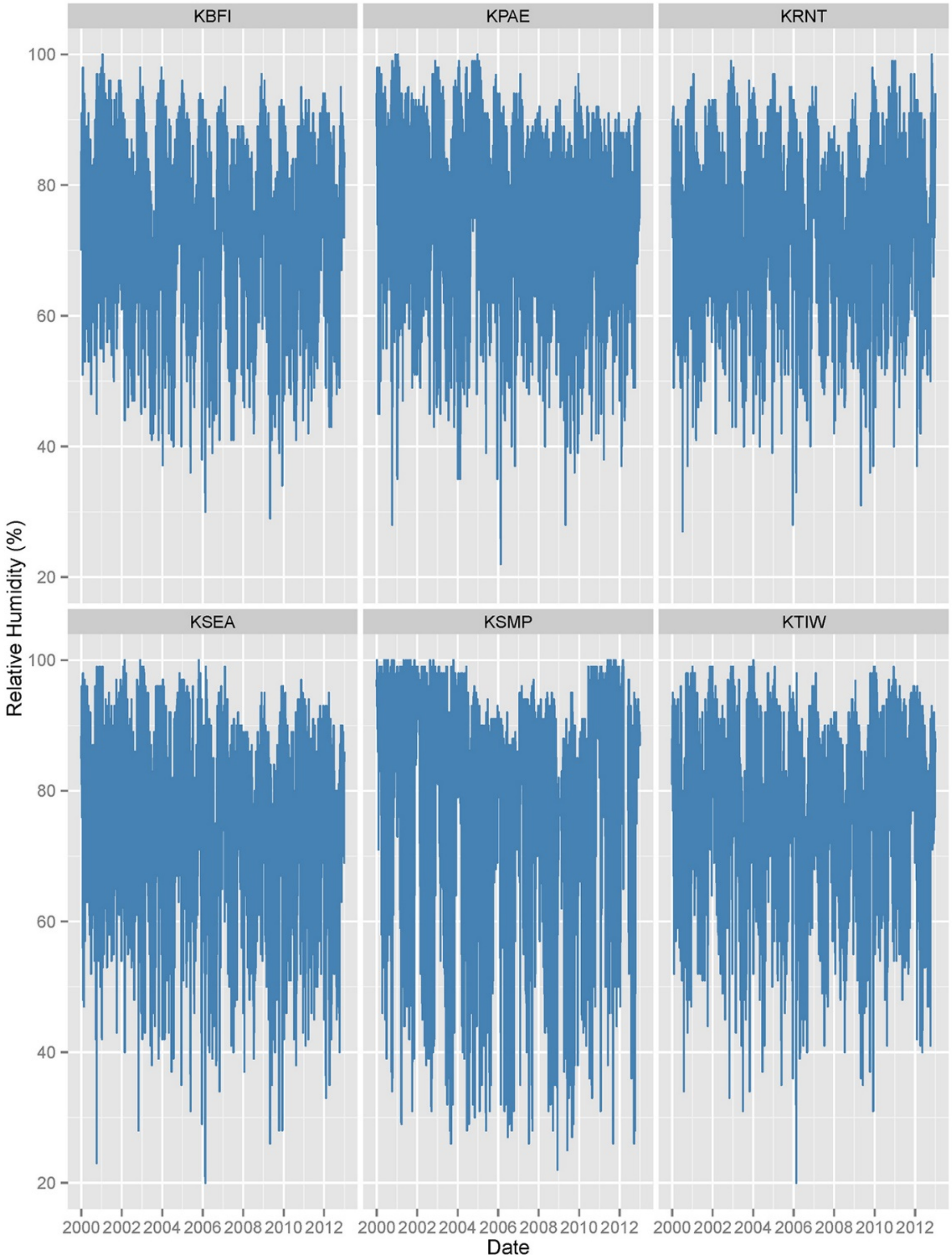


**Appendix Figure 4.** Measured relative humidity by meteorological stations in western Washington State, 2001–2012.

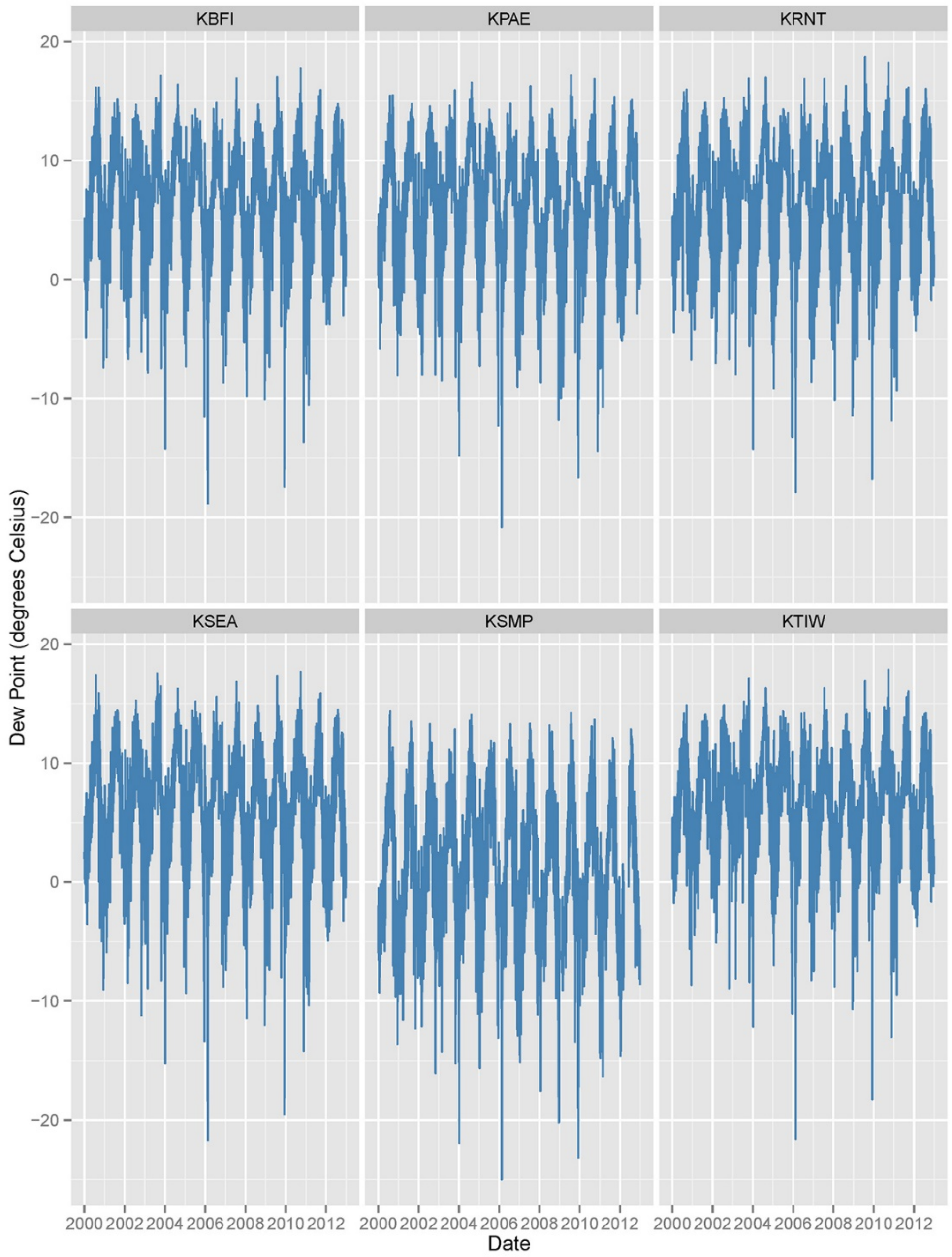


**Appendix Figure 5.** Measured dew point by meteorological stations in western Washington State, 2001–2012.

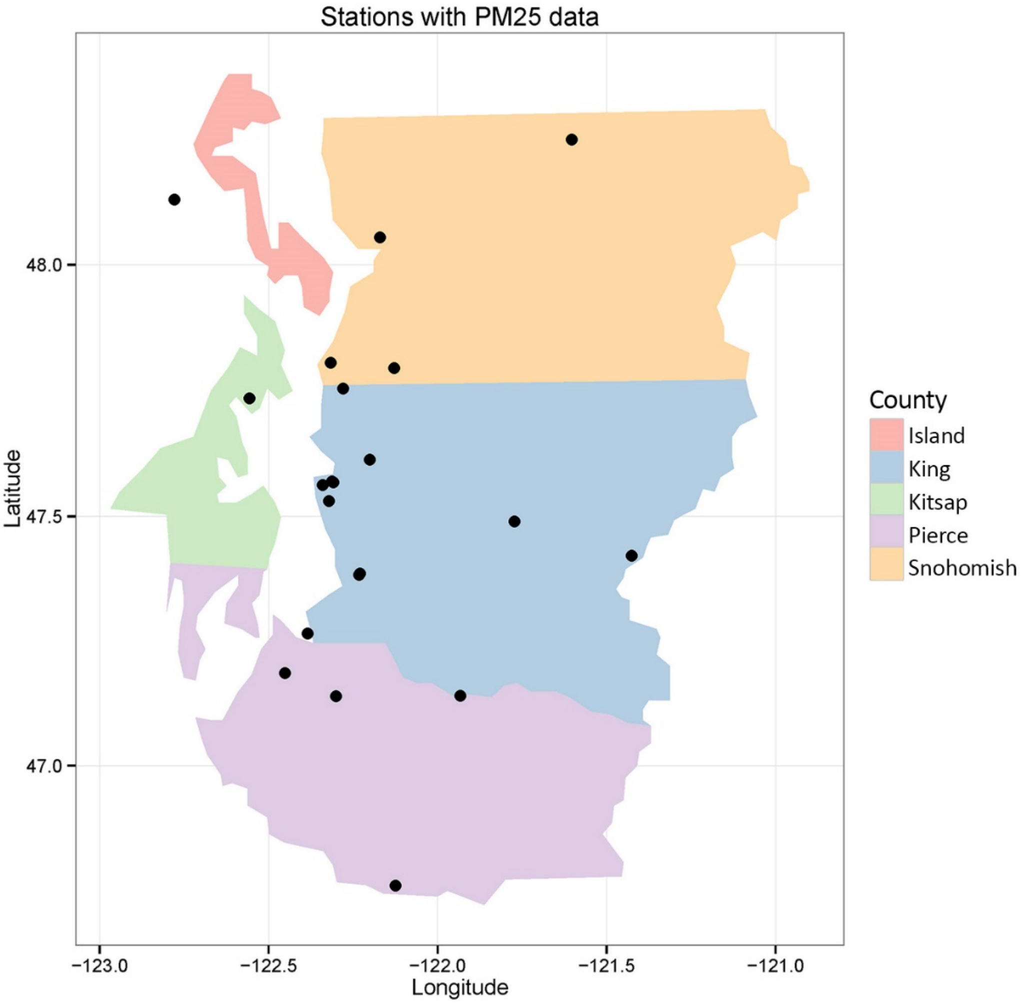


**Appendix Figure 6.** Air quality system (AWS) stations collecting $PM_{2.5}$ data in western Washington State, 2001–2012.

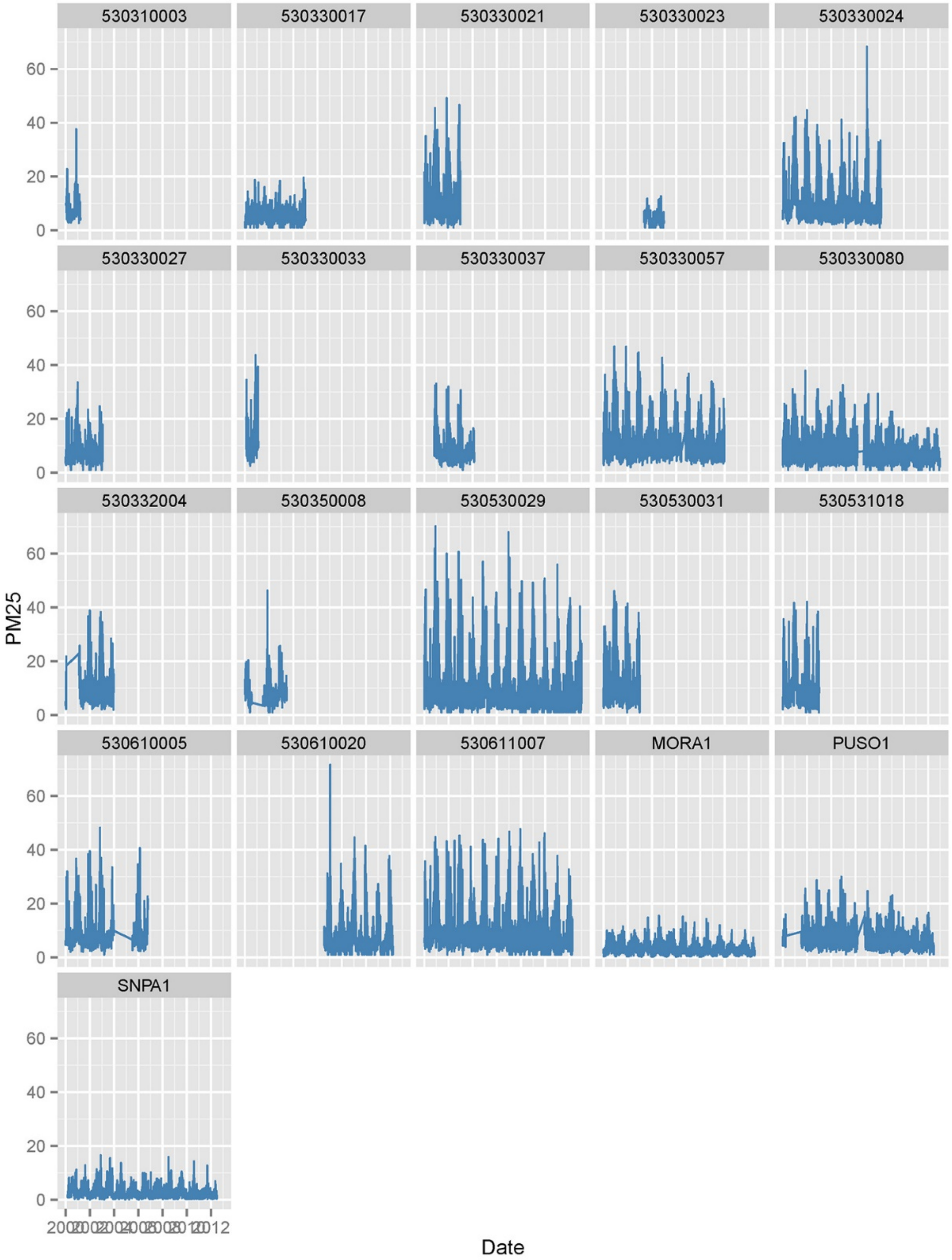


**Appendix Figure 7.** Measured $PM_{2.5}$ by air quality stations in western Washington State, 2001–2012.

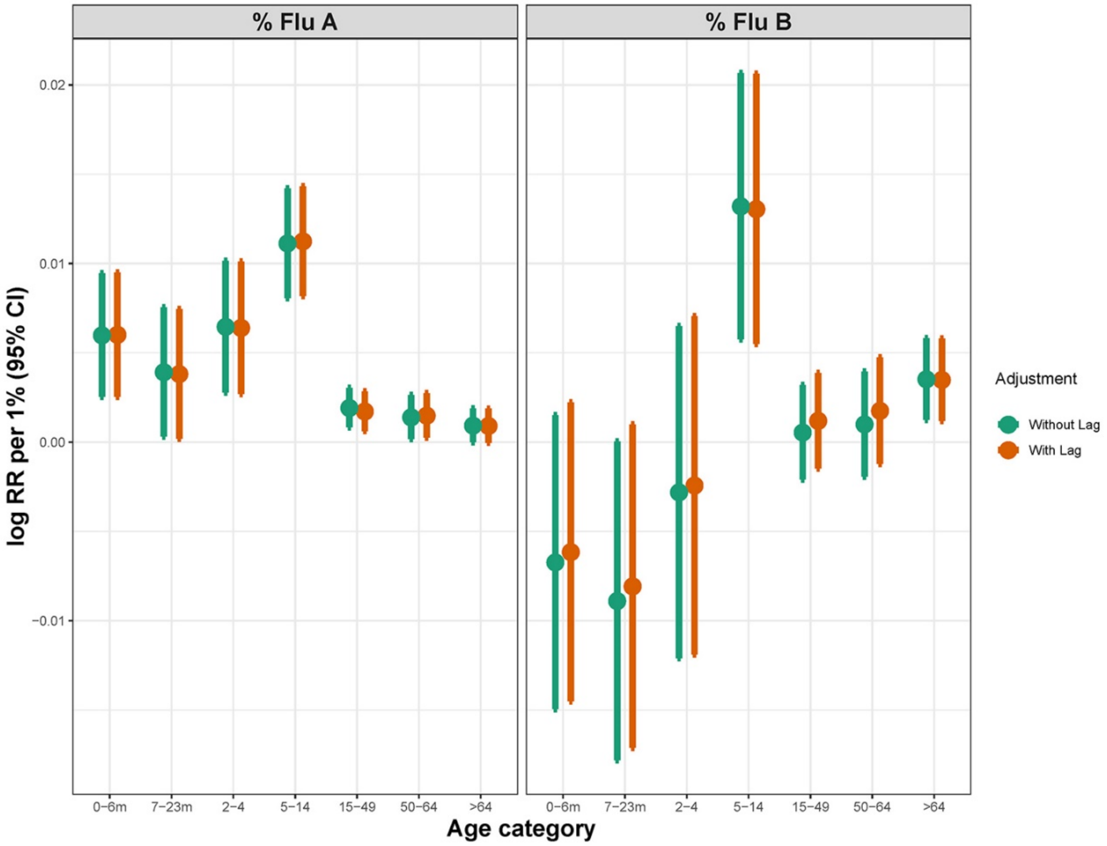


**Appendix Figure 8.** Sensitivity analysis for the expanded models of influenza A– and B–associated hospitalization risk by age with and without lag adjustment for the environmental covariates, western Washington State, 2001–2012.

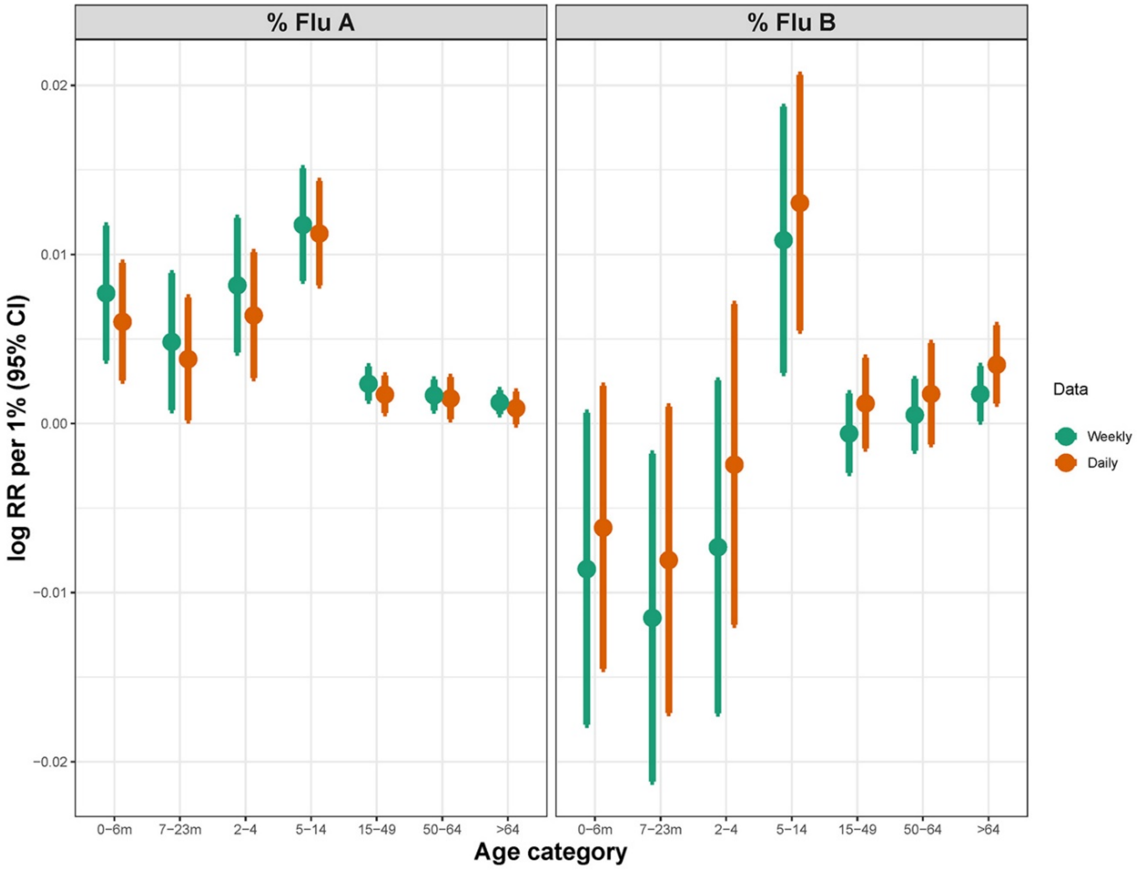


**Appendix Figure 9.** Sensitivity analysis for expanded models of influenza A– and B–associated hospitalization risk comparing analyses using daily vs. weekly events, western Washington State, 2001–2012.
